# Switching to nevirapine (NVP) significantly increases high-density lipoprotein cholesterol (HDL-C) in treatment-experienced patients (NEVICOR study)

**DOI:** 10.1186/1758-2652-13-S4-P79

**Published:** 2010-11-08

**Authors:** JM Rodriguez, M Delgado, P Viciana, MA Lopez-Ruz, M Leal, F Alcacer, E Deig, A Antela, E Pedrol, S Moreno

**Affiliations:** 1 Hospital Ramon y Cajal, Infectious Diseases, Madrid, Spain; 2 Hospital Carlos Haya, Infectious Diseases, Malaga, Spain; 3 Hospital Virgen del Rocio, Infectious Diseases, Sevilla, Spain; 4 Hospital Virgen de las Nieves, Infectious Diseases, Granada, Spain; 5 Hospital Clinico, Infectious Diseases, Valencia, Spain; 6 Hospital General de Granollers, Infectious Diseases, Barcelona, Spain; 7 Hospital Clinico Universitario, Infectious Diseases, Santiago, Spain; 8 Hospital de Sant Pau I Santa Tecla, Infectious Diseases, Tarragona, Spain

## Background

A strong inverse relationship between the plasma concentration of HDL-C and the incidence of coronary heart disease is widely accepted. Few interventions have succeeded to increase plasma HDL levels in HIV-infected pts. NVP has a favorable lipid profile, and clinical trials have suggested that it could have a differential effect on plasma HDL-C levels.

## Methods

Prospective, single arm, multicenter study. We included patients on stable antiretroviral therapy with HIV RNA ≤50 copies/mL for at least one year that were switched to a NVP-containing regimen. Patients receiving lipid lowering therapy were excluded. HDL-cholesterol and other lipid parameters at baseline and after 24 weeks of treatment with NVP are compared.

## Results

Among 130 pts included in the study, 119 (91%) could be evaluated. BL characteristics: median age 44, female 24%, current smokers 53%. Previous AIDS 29%, CD4 count 502/mm^3^. Time on ARV therapy 42 months. Previous therapy: efavirenz 38%, 3 NRTI 12%, PI 50%. Accompanying nucleosides were tenofovir/emtricitabine in 69%, and abacavir/lamivudine in 31%.

Table 1 shows the 24-week results of lipid profile.

**Table 1:** 

		Mean	S. D.	N	p
Triglycerides (mg/dl)	Previous treatment	213.8	178.3	119	

	Nevirapine	154.6	95.3	119	<0.05

TC (mg/dl)	Previous treatment	203.6	48.0	119	

	Nevirapine	198.1	40.5	119	0.108

HDL-C (mg/dl)	Previous treatment	43.8	14.6	119	

	Nevirapine	49.3	16.8	119	<0.05

TC/HDL-C	Previous treatment	5.1	1.9	119	

	Nevirapine	4.3	1.3	119	<0.05

LDL (mg/dl)	Previous treatment	120.2	38.1	107	

	Nevirapine	119.2	32.7	107	0.705

VLDL (mg/dl)	Previous treatment	35.1	19.4	25	

	Nevirapine	23.6	12.6	25	<0.05

At 24 week, the proportion of patients with HDL-C>40 mg/dl were 69.7% (95%CI 60.7-77.8), compared to 52.1% (95%CI 42.8-61.3) before taking NVP (p<0.01). The Framingham risk score decreased from 7.6 to 6.6 (p<0.05) after switching to NVP.

## Conclusions

Switching to NVP-containing regimens in patients on stable therapy is associated with a significant increase in HDL-C and decrease in TC/HDL-c, with an overall improvement in the Framingham score.

